# Supplementary material for: Thermally-nucleated self-assembly of water and alcohol into stable structures at hydrophobic interfaces
Source: Nat Commun. 2016 Oct 7;7:13064. doi: 10.1038/ncomms13064 (PMC5059760; doi:10.1038/ncomms13064)
Supplement: Supplementary Information — Supplementary Figures 1-14 and Supplementary References [file ncomms13064-s1.pdf]

SUPPLEMENTARY FIGURES

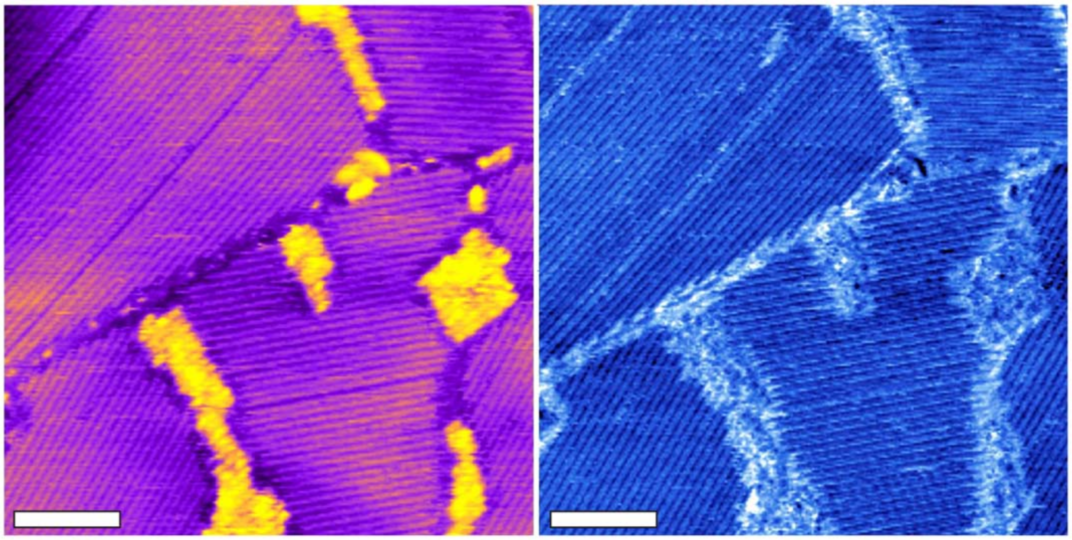

Supplementary Figure 1: Low magnification AFM image of the row structure. Topography (left) and phase (right) show domains of parallel rows that span typically a few hundred nanometres. The rows grow epitaxially on the HOPG surface, forming 120° angles between different domains. A second level of rows (appearing yellow in topography) can form on top of the first level at higher temperatures (see also Fig. 4). The scale bar is 50 nm.

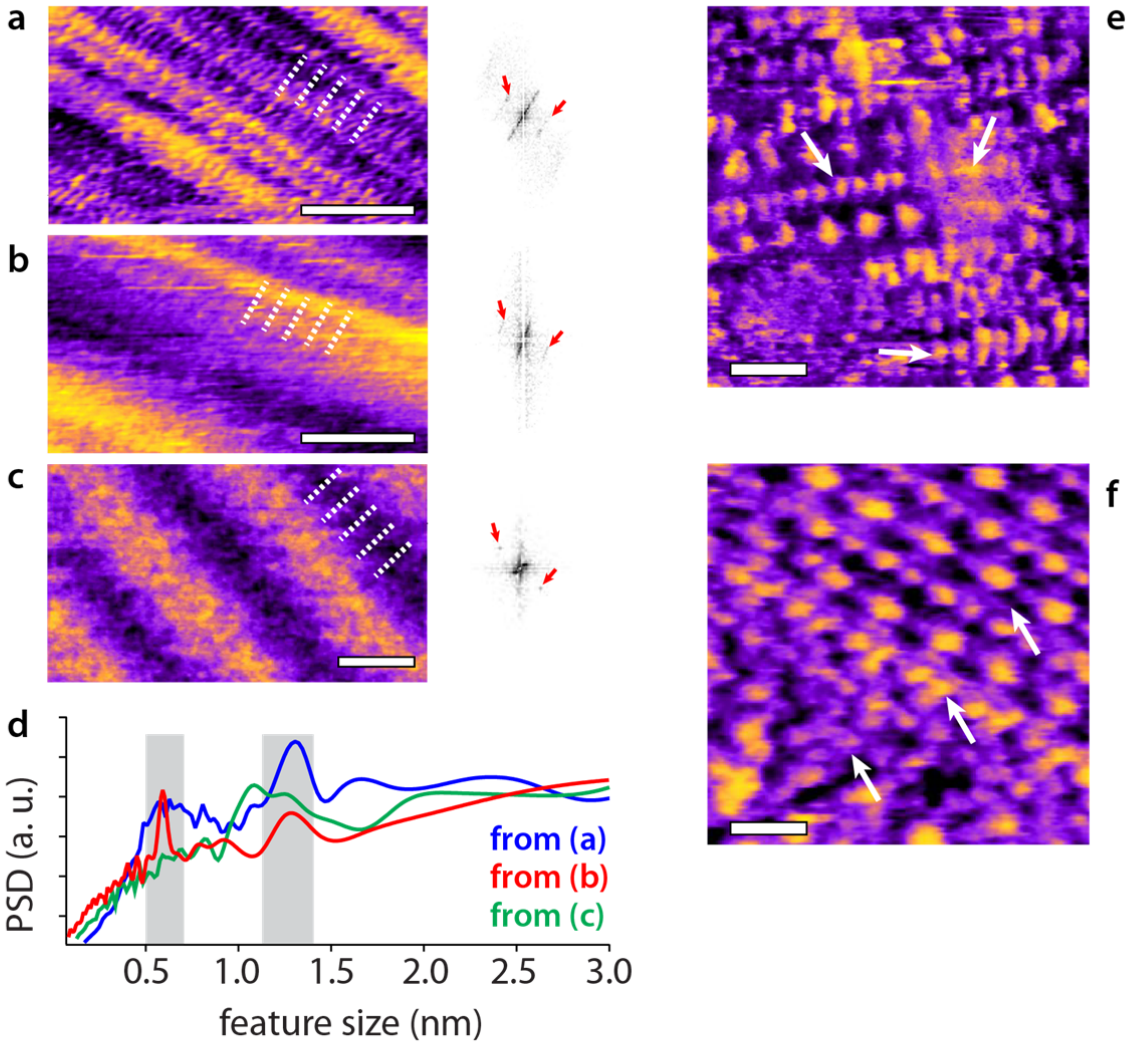

Supplementary Figure 2: High-resolution AFM imaging of different water-alcohol structures at the surface of HOPG in solution. All images are of water-MeOH binary mixtures except (a) where a 1:1 water:MeOH solution has been spiked with a small quantity (<1%) of EtOH (as in Fig. 1a). The Fourier analysis of the most common row-like structure is shown in (a-c) with in each case the original topographic image (purple colour scale) shown on the left, and the Fourier transform (black and white colour scale) shown on the right. The finer structure is highlighted with dashed white lines in topography and the corresponding periodicity and orientation highlighted with red arrows in the Fourier space. Average profiles taken in the Fourier space along the direction corresponding to the smaller features are shown in (d). In most cases, a maximum can be identified around 0.6 nm, corresponding to the size of the smallest features captured by the tip and (a-b). A maximum is also visible at twice that length scale (~1.2 nm), something common when imaging small periodic features with AFM. In (c) the features appear with ~1.2 nm periodicity due to the tip inability to resolve finer details. Each image (a-c) was captured with different tips and on two different AFMs.
At lower MeOH concentration, (here X_{MeOH}~0.05) the interface often shows regular patterns but with a high degree of polymorphism in the molecular arrangements (e-f), suggesting that multiple arrangements are possible and may be competing (white arrows). All AFM images are topographic, with scale bars of 30 Å (a-c), 20 Å (e), and 10 Å (f). The imaging temperatures are 36±3°C in (a-b), 35±0.1°C in (c) and 60±0.1°C in (e-f).

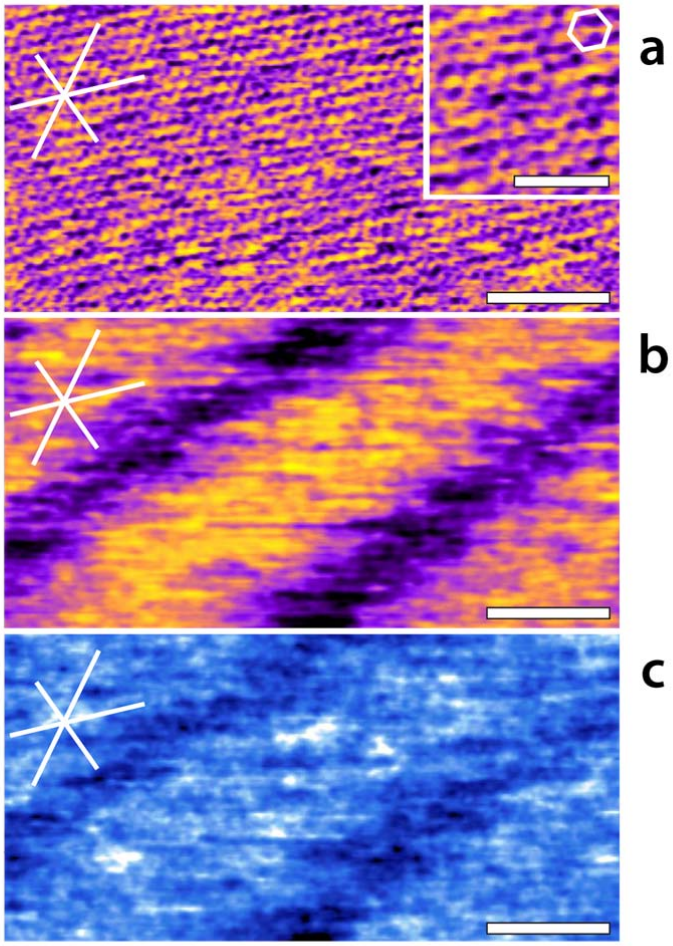

Supplementary Figure 3: Orientation of the rows with respect to the underlying HOPG lattice. An image of the HOPG atomic lattice was obtained in contact mode inside the water-MeOH solution (a). The inset provides a magnified view of the image with the HOPG lattice highlighted. Immediately after, an AM-AFM of the row structure was obtained at the same location with both topography (b) and phase (c). In all images, the crystallographic orientation of the HOPG lattice is represented with white lines, showing that the row grow perpendicularly to main crystallographic directions. The quality of the AM-AFM image is relatively poor due to tip degradation occurring during the scanning in contact. The scale bar is 20 Å in all images expect for the inset in (a) where it is 10 Å.

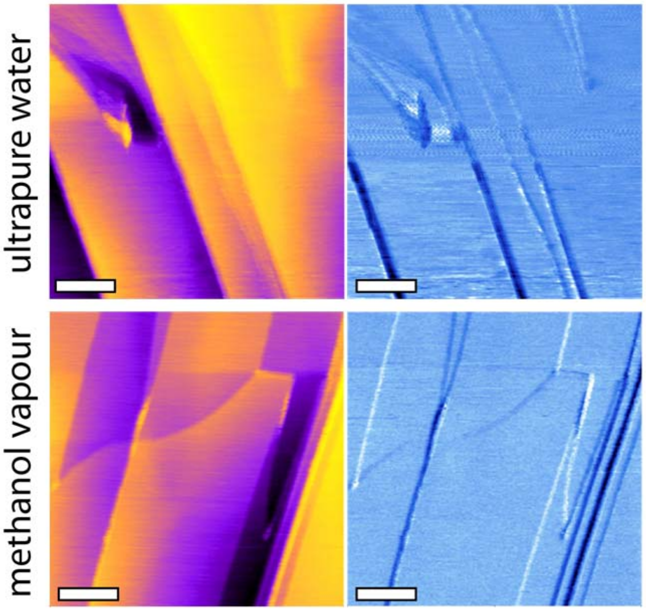

Supplementary Figure 4: Surface of HOPG imaged in pure water (up) and in saturated MeOH vapour (down). As usual, topography is presented left and phase right. The imaging in MeOH where obtained by flushing pure nitrogen inside the AFM cell until the relative humidity level dropped below 2% and a container filled with pure MeOH was then placed inside the cell. The images were taken after the MeOH from the container had evaporated inside the cell. Using this procedure, MeOH covers the surface, as confirmed by Supplementary Fig. 5. The scale bar is 100 nm.

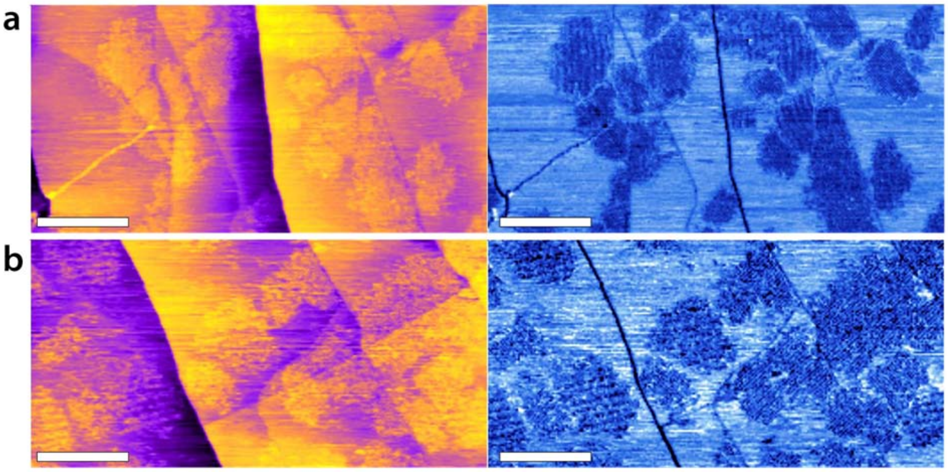

Supplementary Figure 5: Surface of HOPG imaged in pure water after diffusion of MeOH vapour. Topography is presented on the left of the figure and phase on the right. The images were acquired sequentially over a same region of the sample with a 13 min interval between (a) and (b). The domains appear more marked and wider in (b). The scale bar is 200 nm.

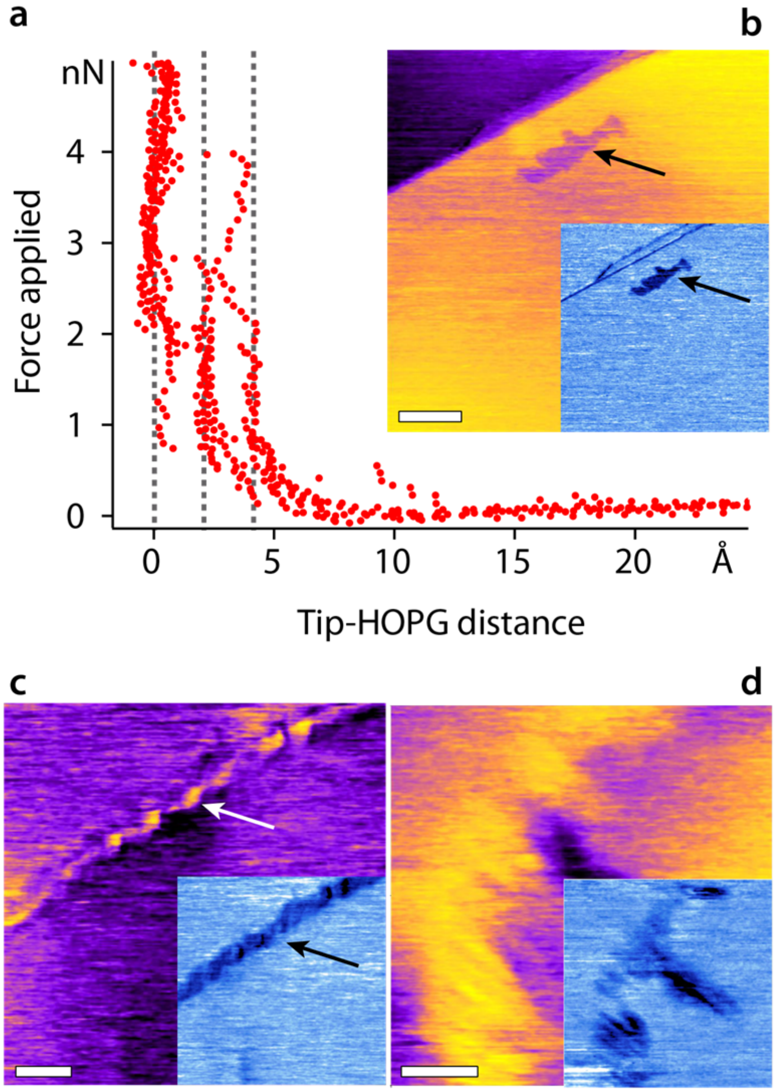

Supplementary Figure 6: AM-AFM images of the interface between HOPG and a 1:1 mixture of water:EtOH at different temperatures. No visible ordered structure could be identified but a cohesive layer is present on the surface already at 30 °C, as identifiable by force spectroscopy (a). At least 2 distinct layers can be identified, each 2.2±0.2 Å thick, consistent with previous reports[1]. The graph superimposes the data from 7 representative curves. At 40 °C, some features can be identified in the layer (black arrow) (b), but no stable molecular details can be obtained and the structures disappear in subsequent images. At 60 °C (c) some stable features are building up close to HOPG's atomic steps with further stable structures visible at 70 °C (d). In all cases, the phase is shown as inset. We note that although no stable 2D assembly is visible, other concentrations or solution containing different alcohol may be able to form stable structures such as shown in Fig. 1a. Since solutions involving water and short alcohols such as MeOH and EtOH exhibit similar mixing properties in the bulk[2], the formation of interfacial structures is likely dominated by atomistic details of the interface. The scale bars are 50 nm (b), 20 nm (c) and 15 nm (d).

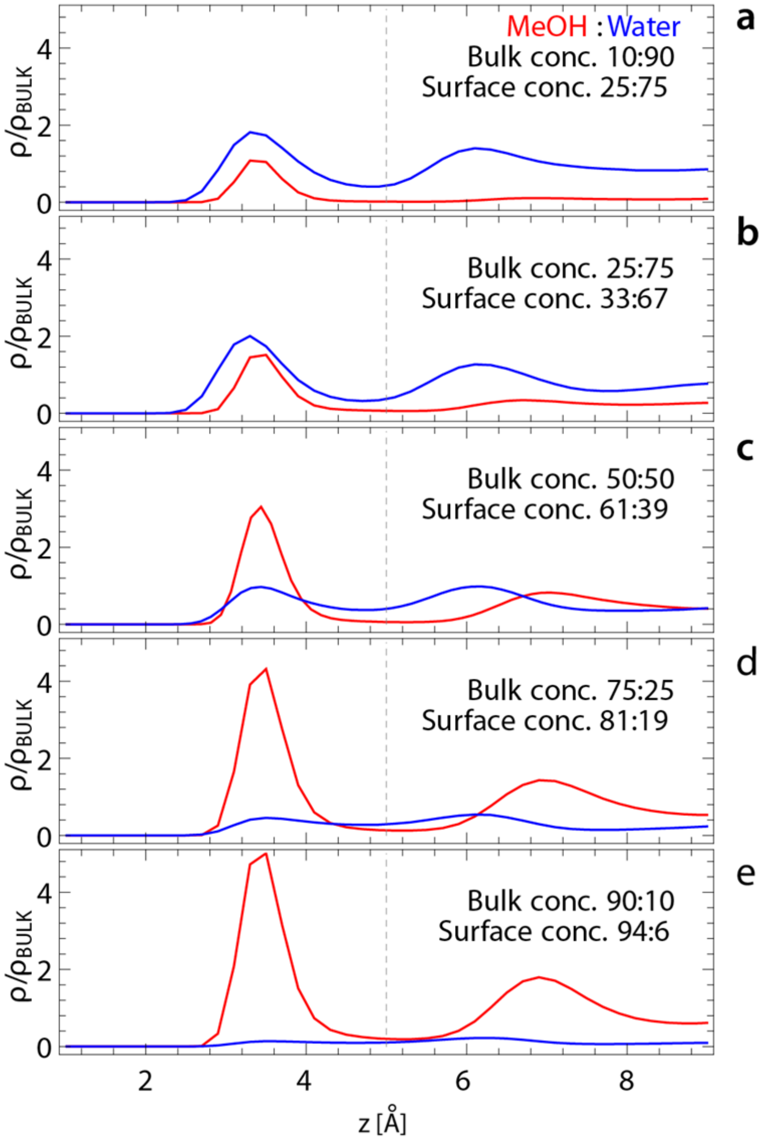

Supplementary Figure 7: Concentration profile of MeOH and water from simulations. The profiles are taken in the z direction (perpendicular to the graphite surface) in water-MeOH mixtures of varying bulk concentration. All simulations were performed using empirical force fields fitted to DFT data, as detailed in the Methods section. We consistently observe an excess of MeOH in the near-surface layer (up to 5 Å from the uppermost layer of graphite) with respect to bulk concentration. In each case, the bulk and effective surface compositions are indicated in the upper right of the figure. The profile in (c) is identical to that shown in Fig. 2b. Given the slow dynamics and the glassy behavior of the solution-HOPG interface, we cannot exclude that the structure and composition we observe correspond to a meta-stable state, acting as the precursor of a structured layer with a precise stoichiometry.

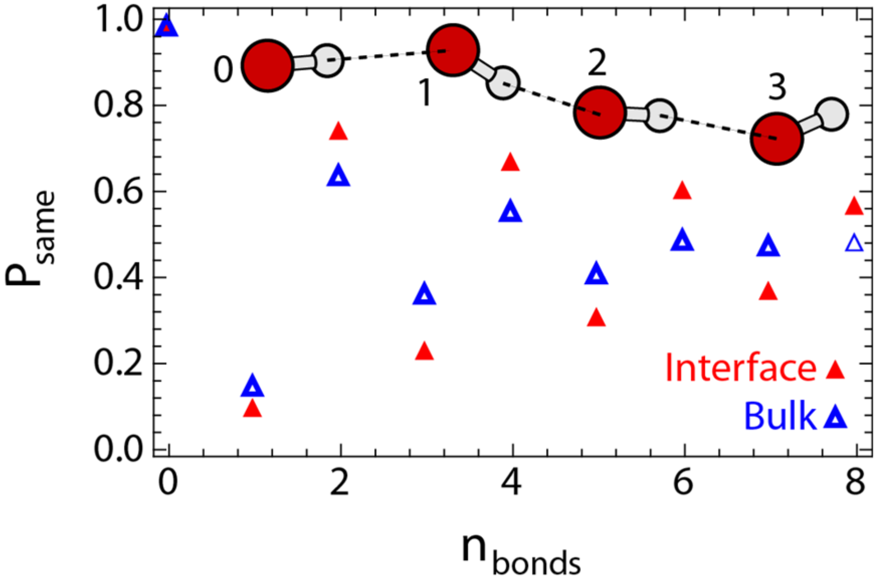

Supplementary Figure 8: Analysis of the topological correlation between MeOH and water molecules in the 1:1 mixture from force field simulations. The graph presents the probability P_{same} of finding a MeOH molecule after a distance of n bonds while moving along the H-bond network. The cartoon shown in inset provides an illustration of the analysed correlation. The probability was determined based on a data-driven, self-consistent definition of the hydrogen bond[3]. The points are shown for both the hydrogen bond network at the interface with HOPG (solid red triangles) and in the bulk liquid (open blue triangles). It is clear from the graph that the most probable arrangement is an alternated network of MeOH and water molecules (every second bond has the highest probability), as previously reported for the bulk solution[2,4,5]. The trend is further reinforced at the interface, with longer-range correlations. The simulations were conducted at 300 °K. Molecules were considered as being at the interface if within 5 Å from the topmost graphite layer, consistent with Fig. 2 and Supplementary Fig. 7.

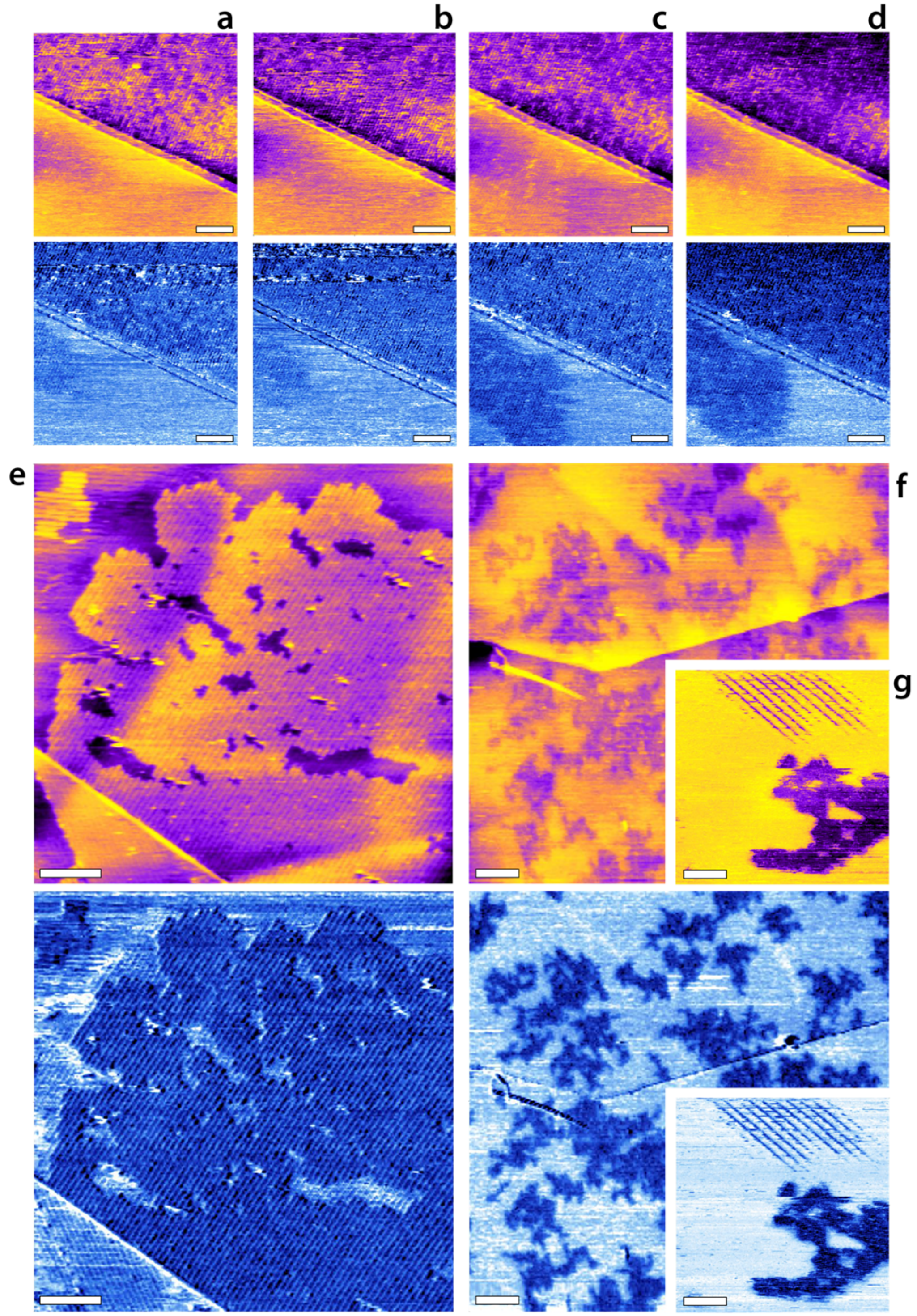

Supplementary Figure 9: Growth of structured interfacial domains driven by temperature as presented in Fig. 4 of the paper. The full set of data is presented here with both phase and amplitude, and in the usual colour scale used throughout the paper. Large amplitude undulations of the HOPG surface can be seen in the topographic image in (e), making the interfacial structures less clear. These are due to stress-induced deformations in the bulk HOPG substrate. The scale bars are 50 nm (a-e), 100 nm (f) and 20 nm (g).

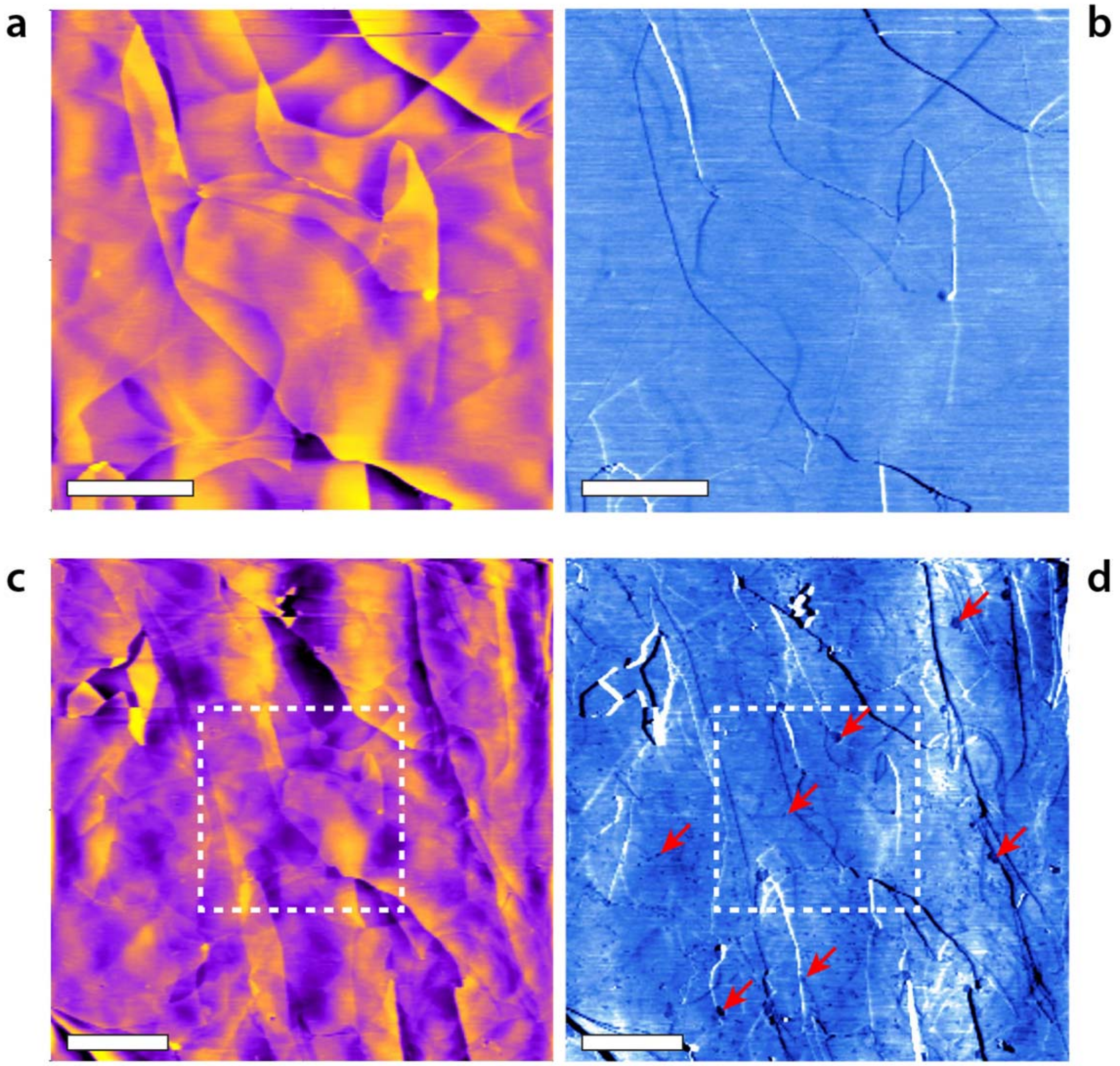

Supplementary Figure 10: Control experiments conducted to rule out tip-induced nucleation in AFM imaging. The experiment was conducted at 35°C in a 1:1 water:MeOH solution. After only 1 minute at 35 °C (heated from 25 °C), no interfacial structure is visible in topography (a) or in phase (b). High-resolution images (10 nm) were then acquired at the centre of (a) during 8 minutes without identifying any notable structure. A large frame acquired subsequently (c-d) revealed numerous nucleation points appearing as darker spots in the phase (arrow in d). The spots are spread evenly across the surface, with no notable difference between the region previously scanned in (a) (inside the dotted square) or never scanned previously (outside of the dotted square). This demonstrates that the tip does not play a role in nucleating the structure, provided the AFM laser does not reach the HOPG surface (see Supplementary Fig. 14). The topographic images have been flattened using a high-order polynomial to compensate for the curviness of the HOPG surface. The scale bars are 500 nm (a-b) and 1 μm (c-d).

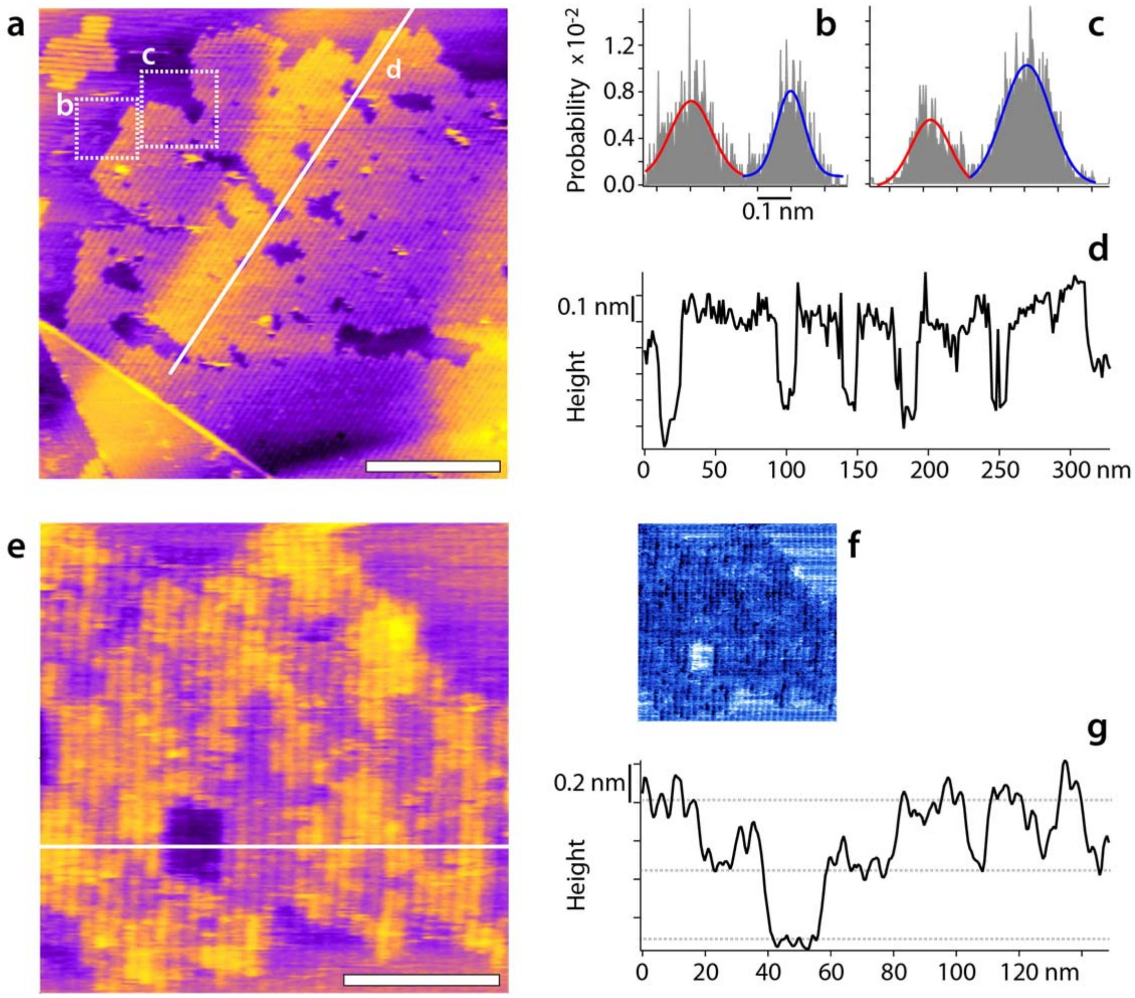

Supplementary Figure 11: Topographic images exemplifying the existence of multiple layers of row-like structures occasionally observed at higher temperatures. An inset showing a portion of Supplementary Fig. 9e (obtained at 45°C) is given in (a). The whole surface is covered with row-like structures with a large central region exhibiting a second layer. The dotted squares, selected at the edge of the second layer, designate the areas used for calculating the height histograms shown in (b) and (c). Due to the natural topographic undulations of the graphite surface, the areas selected are relatively small so as to allow for a reliable quantification of the layer's thickness. In (b) and (c) the first peak (red Gaussian fit) corresponds to the first layer while the second peak (blue Gaussian fit) to the second layer. The derived thicknesses for the second layer (distance between red and blue Gaussian position) are 2.9±0.4 Å (b) and 2.8±0.3 Å (c). A profile taken over most of the second layer (solid white line in (a)) is shown in (d), highlighting gaps in the second layer. The gaps are largely perpendicular to the rows' direction, consistent with the proposed molecular model. At higher temperature (55°C), fragments of a third layer could occasionally be observed (e). The third layer is largely irregular with only small islands visible, suggesting a progressive transition from an order and stable first layer to the bulk liquid. The phase image corresponding to (e) is given in (f). A height profile taken over the 3 layers (solid line in (e)) is given in (g). All images were acquired in a 1:1 water-MeOH solution. The scale bars is 100 nm (a) and 50 nm (e)

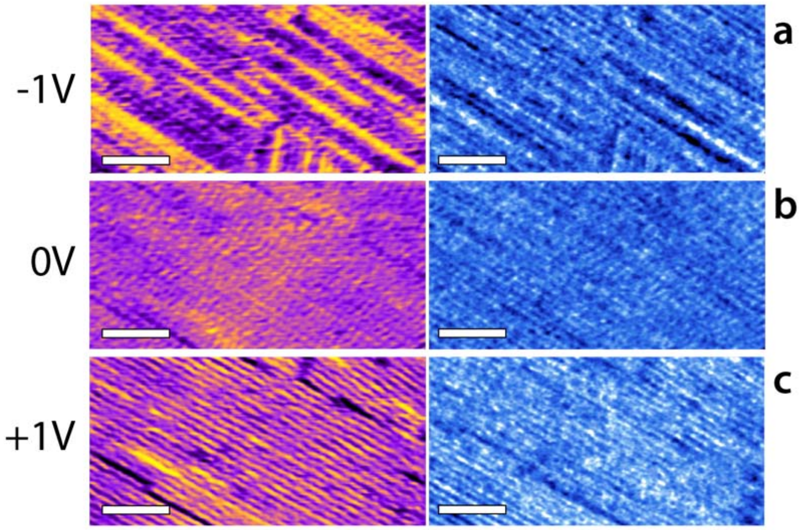

Supplementary Figure 12: Illustration of the robustness of the water-MeOH row structure. Here the structures are exploited to template ionic rows using a trans-interface electric field (a-c). The potential indicated is the voltage applied to the HOPG substrate with respect to a counter electrode located in the solution, which contains 4mM of dissolved RbCl. At -1V (a) Rb^+ cationic ‘wires’ are visible both in topography and phase. The rows disappear when the voltage is set to 0V (b). Reversing the voltage (c) creates a different assembly, presumably involving Cl^- anions. Images (a-c) are taken sequentially in a same location. It was not possible to determine the atomistic details of these ionic structures from AFM images, a task beyond the scope of this paper. Additionally, ion-specific hydration effects[6] are likely to play a role in the assembly. Nonetheless, this simple experiment illustrates the robustness of the interfacial assembly and some of the possibilities offered by the interfacial structures as template for directed nanoscale self-assembly. Control experiment involving only monovalent salt in pure water did not show any stable or ordered organisation of the adsorbed ions on HOPG (Supplementary Fig. 12). The scale bar is 40 nm.

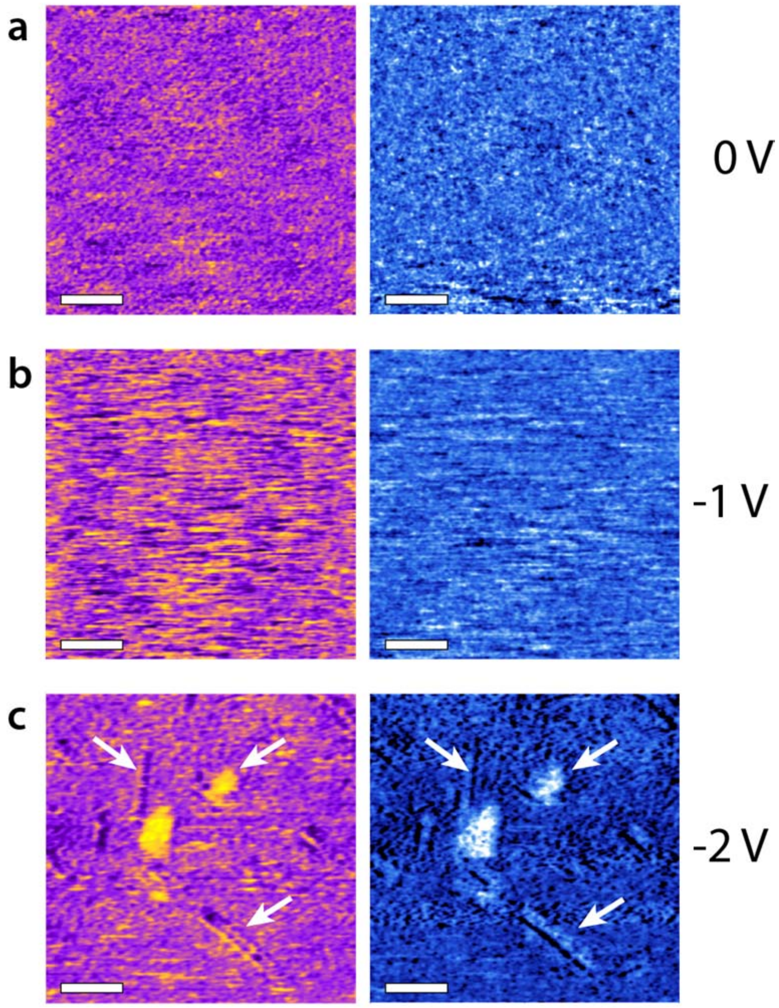

Supplementary Figure 13: Control AFM experiment examining the adsorption of monovalent metal ions at the surface of HOPG in pure water. The setup used is identical to that used in Fig. 5. When no voltage is applied, the HOPG surface appears smooth with no obvious feature (a). At -1V (b), the surface shows increases roughness, presumably due to the adsorption of ions, but no stable structure can be identified. At even higher voltages (c), electrochemical reactions occur leaving deposits on the surface and highlighting surface features in registry with the HOPG lattice (arrows). In order to favour the adsorption of ions on the surface, a higher concentration was used than in Fig. 5 (10 mM). The experiment was conduced in KCl for the near-identical hydration properties of K^+ and Rb^+ (also at charged interface[6]) but the higher charge density of K^+ which should favour adsorption under an electrical potential. The scale bar is 50 nm in all images. The colour scales represents height variations of 4 Å (a), 6 Å (b) and 8 Å (c), and phase variations of 5° (a), 10° (b), and 15° (c).

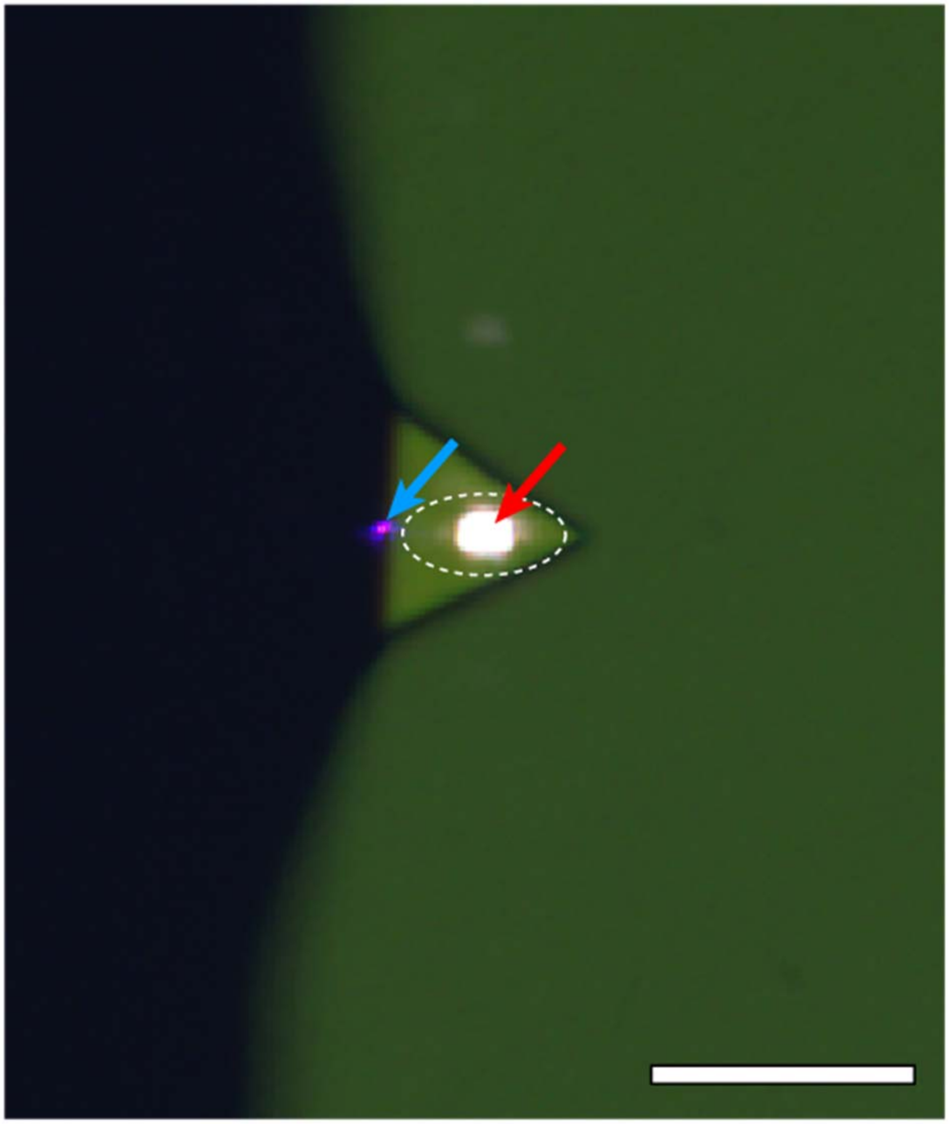

Supplementary Figure 14: Top view of the Cypher AFM triangular cantilever (ArrowUHF-AUD) with the laser configuration used for most of the experiments. A small laser spot (3µm by 9µm, λ=850 nm) was used to detect any displacement of the cantilever (red arrow) instead of a standard laser (10µm by 30µm, dotted ellipse) to avoid diffraction on the substrate from the edges of the cantilever. Use of the standard spot yielded unreliable results with temperature, presumably due to laser-induced local heating of the HOPG. The blue laser used for the photothermal excitation is also partly visible (blue arrow). The scale bar is 50 µm.

SUPPLEMENTARY REFERENCES

1. Kanda, Y., Nakamura, T. & Higashitani, K. AFM studies of interaction forces between surfaces in alcohol–water solutions. *Coll. Surf. A* **139,** 55–62 (1998).
2. Dixit, S., Crain, J., Poon, W. C. K., Finney, J. L. & Soper, A. K. Molecular segregation observed in a concentrated alcohol–water solution. *Nature* **416,** 829–832 (2002).
3. Gasparotto, P. & Ceriotti, M. Recognizing molecular patterns by machine learning: An agnostic structural definition of the hydrogen bond. *J. Chem. Phys.* **141,** 174110 (2014).
4. Batista da Silva, J. A., Moreira, F. G. B., Leite dos Santos, V. M. & Longo, R. L. On the hydrogen bond networks in the water–methanol mixtures: topology, percolation and small-world. *Phys. Chem. Chem. Phys.* **13,** 6452–6461 (2011).
5. Bakó, I., Megyes, T., Bálint, S., Grósz, T. & Chihaia, V. Water–methanol mixtures: topology of hydrogen bonded network. *Phys. Chem. Chem. Phys.* **10,** 5004–5011 (2008).
6. Ricci, M., Spijker, P. & Voitchovsky, K. Water-induced correlation between single ions imaged at the solid–liquid interface. *Nat. Commun.* **5,** 4400 (2014).
